# Cancer Risk in Mothers of Men Operated for Undescended Testis

**DOI:** 10.1371/journal.pone.0014285

**Published:** 2010-12-10

**Authors:** Hadriano M. Lacerda, Lorenzo Richiardi, Andreas Pettersson, Marine Corbin, Franco Merletti, Olof Akre

**Affiliations:** 1 Cancer Epidemiology Unit, CPO and CeRMS, University of Turin, Italy; 2 Clinical Epidemiology Unit, Karolinska Institutet, Stockholm, Sweden; 3 Centre for Public Health Research, Massey University Wellington Campus, Wellington, New Zealand; Alberta Research Centre for Health Evidence, University of Alberta, Canada

## Abstract

**Background:**

Undescended testis, or cryptorchidism, occurs in 2–5% of boys born at term, and by 12 months of age about 1% of all boys have manifest cryptorchidism. Several hormonal substances control this process and disruption of the foetal sex-hormones balance is a potential cause of undescended testis, however, to a great extent the aetiology of cryptorchidism is unclear.

**Methodology:**

To study risk factors involved in the aetiology of undescended testis, we assessed cancer risk in 15,885 mothers of men operated for undescended testis in Sweden. Women were followed-up for a median period of 23 years during which 811 first primary malignancies occurred. Their cancer incidence was compared with that in the general population estimating standardized incidence ratio (SIR) and corresponding 95% confidence interval (CI).

**Principal Findings:**

The overall cancer risk experienced by the mothers of cryptorchid men did not differ significantly from that of the general population (SIR = 0.94; 95% C.I. = 0.88–1.01). Specifically, there was a reduction in ovarian cancer risk (SIR = 0.72; 95% C.I. = 0.51–0.99), while the risk of lung (SIR = 1.38 95% C.I. 1.03–1.81) and biliary tract/liver cancer (SIR: 1.76, 95% CI: 1.03–2.82) were increased.

**Conclusions:**

Although we cannot rule out the role of chance, our data suggest a positive association between undescended testis and maternal lung cancer and a negative association with ovarian cancer, where the first may be partly attributable to smoking and the second to an altered hormonal milieu during pregnancy and thus both exposures may be risk factors for cryptorchidism.

## Introduction

Undescended testis, or cryptorchidism, occurs in 2–5% of boys born at term [1]. Testes that are undescended at birth may descend spontaneously during early life, but by 12 months of age about 1% of all boys have manifest cryptorchidism [1], [2].

During male foetal development, the testes descend into the scrotum in two phases, the transabdominal followed by the inguinoscrotal phase. Several hormonal substances control this process, but androgens are known to be instrumental in the second phase of testis descent [3]. It has therefore been suggested that disruption of the foetal sex-hormones balance is a potential cause of undescended testis. Prematurity and low birth weights are associated with an increased prevalence of cryptorchidism [4], [5], [6], as well as placental dysfunction and maternal smoking have been indicated as other potential risk factors [6], [7], [8]. To a great extent, however, the aetiology of cryptorchidism is unclear.

To find further clues on possible risk factors involved in the aetiology of undescended testis, we have analyzed maternal cancer risk as a proxy to foetal exposure in a large cohort of cryptorchid men.

## Results

The cohort consisted of 15885 women followed-up for a median period of 23 years (range: 0–36 years), during which 811 first primary malignancies, 29 second-primary malignancies and 1 third-primary malignancy occurred. The main characteristics of the cohort are summarised in Table 1.

**Table 1:** Characteristics of the cohort of mothers of cryptorchid boys.

Characteristic	N° of subjects (N = 15885)	Person-years
Age (years)		
18–24	5432	132356
25–29	3679	127231
30–34	3306	69662
35–39	1230	25781
40–44	228	4869
45–49	10	229
Calendar period		
1965–1969	2767	89599
1970–1974	3572	98907
1975–1979	3444	79606
1980–1984	2935	53597
1985–1989	2206	30048
1990–1994	900	8047
1995+	61	323

The overall cancer risk experienced by the mothers of cryptorchid men did not differ significantly from that of the general population (SIR = 0.94; 95% C.I. = 0.88–1.01) (Table 2). Specifically, there was an increased risk of biliary tract/liver (SIR = 1.76 95% C.I. 1.03–2.82) and lung (SIR = 1.38 95% C.I. 1.03–1.81) cancers and a reduction in ovarian cancer risk (SIR = 0.72; 95% C.I. = 0.51–0.99). None of the associations was substantially modified by age at diagnosis (Table 3) or the exclusion of the mothers of men born before 1970 (SIR of biliary tract/liver cancer: 1.86, 95% CI: 0.89–3.43; SIR of lung cancer: 1.38, 95% C.I. 0.94–1.95 cancers; SIR of ovarian cancer: 0.73, 95% C.I. 0.47–1.08).

**Table 2:** Standardized incidence ratios (SIRs), and corresponding 95% confidence intervals (CI) of cancer among mothers of cryptorchid boys.

Cancer site	ICD-7 Code	Obs.	Exp.	SIR	95% CI
All sites	140–209,excl. 191	811	859.69	0.94	(0.88–1.01)
Oral cavity, pharynx	140–148excl. 142,146	5	7.74	0.65	(0.21–1.51)
Stomach	151	6	11.54	0.52	(0.19–1.13)
Colon-rectum	153, 154	43	53.76	0.80	(0.58–1.08)
Biliary tract, liver	155	17	9.66	1.76	(1.03–2.82)
Pancreas	157	6	10.24	0.59	(0.21–1.28)
Lung	162	52	37.60	1.38	(1.03–1.81)
Breast	170	325	335.14	0.97	(0.87–1.08)
Cervix uteri	171	48	53.78	0.89	(0.66–1.18)
Corpus uteri	172	38	38.12	1.00	(0.71–1.37)
Ovary	175	38	52.75	0.72	(0.51–0.99)
Kidney	180	19	12.87	1.48	(0.89–2.31)
Urinary organs	181	8	11.32	0.71	(0.31–1.39)
Skin malignant melanoma	190	63	56.99	1.10	(0.85–1.41)
Nervous system	193	38	43.17	0.88	(0.62–1.21)
Thyroid gland	194	16	18.90	0.85	(0.48–1.37)
Endocrine glands	195	23	25.51	0.90	(0.57–1.35)
Non-Hodgkin's lymphoma	200	20	19.19	1.04	(0.64–1.61)
Leukemias	204	14	16.30	0.86	(0.47–1.44)
Unspecified Sites	199	17	18.60	0.91	(0.53–1.46)

Note: SIR: Standardized Incidence Ratio; CI: Confidence Interval; Obs.: Observed; Exp.: Expected.

**Table 3:** Age-specific standardized incidence ratios (SIRs) and 95% confidence intervals (CIs) of biliary tract/liver, lung and ovarian cancer among mothers of cryptorchid boys.

Age groups (years)*	Observed	SIR	95% CI
Biliary tract/liver cancer			
<45	4	1.99	(0.54–5.10)
45–54	7	1.68	(0.68–3.46)
55+	6	1.72	(0.63–3.75)
Lung cancer			
<50	22	1.47	(0.92–2.22)
50–59	19	1.12	(0.68–1.75)
60+	11	1.93	(0.96–3.46)
Ovarian cancer			
<45	14	0.71	(0.39–1.20)
45–54	16	0.69	(0.40–1.13)
55+	8	0.79	(0.34–1.56)

*Due to a different age-distribution in incidence age cut-offs differed for lung cancer.

## Discussion

In this cohort study, we found that mothers of men operated for undescended testis have an increased risk of developing cancer of the biliary tract/liver and lung, and a decreased risk of developing ovarian cancer.

We used strict criteria for the identification of men with cryptorchidism (based both on diagnosis and surgical procedure), thus maximizing specificity of the exposure status. In addition, the use of highly complete nation-wide registries enabled the identification of 99% of the mothers and a complete follow-up. It should be noted, however, that since we analysed the risk of several different cancer sites (n = 19) some of our results might be explained by chance alone. Moreover, the comparison of the cancer incidence between the mothers of cryptorchid men and the general Swedish population is biased by the fact that only the latter includes nulliparous women. If women with children have, on average, different health status, lifestyle and socio-economic conditions than nulliparous women, this biased comparison may alter the finding of no overall difference in cancer risk among mothers of cryptorchid men. In addition, the bias may affect our results on breast and ovarian cancer, which are both inversely associated with number of children [9], [10]. However, while the risk was slightly decreased for breast cancer it was strongly reduced for ovarian cancer, suggesting that the results on ovarian cancer are not entirely explained by this source of bias. Indeed the bias resulting by the inclusion of childless women in the reference population is expected to be rather limited. For example, assuming that i) 10% of the women in the Swedish population remain childless, ii) these women have a 40% increased risk of breast cancer and iii) the risk of breast cancer is the same among mothers of cryptorchid boys and the general population, our study would find an SIR 0.98 (instead of 1.0) due to the bias introduced by parity.

The pathogenesis of ovarian cancer is largely unknown. It appears to be strongly influenced by hormonal factors [11], as for instance, hormonal replacement therapy has been associated with an increased risk of ovarian cancer [12], while contraceptive therapy and multi-parity are documented protective factors against this cancer [11]. As sex hormones levels during pregnancy are also implicated in the aetiology of cryptorchidism [13] our finding of a 30% reduction in risk of ovarian cancer among mothers of men with cryptorchidism is intriguing. Recently, among term deliveries, a strong protective effect of low birth weight on the maternal risk of epithelial ovarian cancer has been found, whereas birth weight seemed to have little effect on the risk of ovarian cancer among preterm births [14], [15]. This association was interpreted as a sign of altered hormonal milieu during pregnancy. Interestingly these factors, namely hormonal patterns during pregnancy, prematurity and low birth weight have all been linked to crytporchidism [6], [16].

Our finding of an increased risk of lung cancer in mothers of cryptorchid men may be explained by shared risk factors, since maternal smoking during pregnancy has been reported to be associated with an increased risk of cryptorchidism in some studies [5], [6], [8], although not all [16], [17]. However, apart from lung cancer, we did not find among the mothers of cryptorchid boys increased risks of other smoking-related cancers, such as head and neck cancer and bladder cancer. Interestingly, maternal lung cancer has been associated also with the risk of testicular cancer in a number of studies [18], [19], [20] but maternal smoking has been repeatedly found not to be associated with testicular cancer risk [21]. Yet unidentified mechanisms should thus explain the link between lung cancer in mothers and testicular cancer in sons [22] and similar mechanisms could also have a role in explaining the association between maternal lung cancer and cryptorchidism.

The increased risk of cancer of the liver/biliary tract that we found was unexpected and in the lack of an a priori hypothesis and a plausible post hoc-explanation, we consider chance as the most likely explanation.

In conclusion, our data suggest a positive association between undescended testis and maternal lung cancer that may be partly attributable to smoking. The inverse association between cryptorchidism and maternal ovarian cancer merits replication, as it is consistent with potential risk factors for cryptorchidism, including an altered maternal hormonal milieu during pregnancy.

## Methods

The study population was identified through a linkage of several Swedish data sources using the national registration number (NRN). All Swedish residents alive in 1947 onward have been assigned a 10-digit NRN, which is a unique personal identifier referred to in all medical records and official registers. Through the use of the NRN, it is possible to link information from several databases together.

Since 1958 in Sweden, physicians as well as pathologists, or cytologists, who confirm them, must report all newly diagnosed malignant tumours to the National Cancer Registry (The National Board of Health and Welfare, Swedish Cancer Registry: http://www.social-styrelsen.se/en/Statistics/statsbysubject/CancerRegistry.htm). Completeness of cancer registration is estimated above 95% [23].

In 2000, Statistics Sweden began a linkage between the several data sources from the national registration and created the Swedish Multi-Generation Register, which contains information on the parents of all individuals in Sweden born in 1932 onward and survived until 1961. Adoption or other non-biologic relations are flagged in the register [24].

We have previously described the cohort of men operated at different ages for cryptorchidism, assembled using the Swedish Hospital Discharge Register [25]. By linking this cohort of men (n = 17396 for the present study) to the Swedish Multi-Generation Register, we identified their mothers. The linkage was 99% complete (250 missing), resulting in a cohort of 16864 mothers (272 mothers had two cryptorchid sons and 5 mothers had 3 cryptorchid sons). Information on migration obtained from the Registry of Population and Population Changes was used to exclude 747 women who were not resident in Sweden when they delivered their son. Finally, we excluded 232 women aged less than 18 at the time of delivery from the cohort, leaving 15,885 women for analysis.

The mothers were followed-up from the date of delivery of their cryptorchid son to the date of emigration, death, or December 31, 2000, whichever occurred first. Information on cancer incidence was obtained from the Swedish Cancer Registry. Coding of multiple primary cancers followed a common set of rules proposed by the International Association of Cancer Registries and the IARC [26]. According to these rules, a primary cancer is one that originates in a primary site or tissue and is thus not an extension, a recurrence, or a metastasis.

For all malignancies combined (excluding non-melanoma skin cancers) and for each cancer site with at least 10 observed cases, we calculated the standardized incidence ratio (SIR) comparing the number of observed cases with the number of expected cases on the basis of the 5-year age and period-specific rates in the Swedish population. We estimated 95% confidence intervals (C.I.) assuming a Poisson distribution and carried out the analyses using the software STATA®, version 9.

For cancer sites associated with a statistically significant SIR, we carried out further analyses stratifying by attained age. We also checked the effect of the incomplete coverage of the Swedish population by the Hospital Discharge Register by sensitivity analysis excluding 2767 mothers of boys born before 1970, when the Register was less complete.
